# Animal models of ectodermal dysplasia

**DOI:** 10.1186/1746-160X-8-S1-I9

**Published:** 2012-05-25

**Authors:** Tosso Leeb

**Affiliations:** 1 Institute of Genetics and DermFocus, Vetsuisse Faculty, University of Bern, Switzerland

## 

Various forms of ectodermal dysplasia (ED) have been identified in animals. These animal models of ED may help in our understanding of the pathogenesis of ED and the development of novel therapeutic approaches. Mice, dogs, and cattle with mutations in the X-linked *EDA* gene have been reported and show clinical features that closely resemble X-linked hypohidrotic ectodermal dysplasia in humans. We still do not completely understand the complex signaling pathways, which are required for the normal development of the various ectodermal appendages. Spontaneous animal mutants offer the chance to identify additional components of this complex regulatory network. One such example is represented by the hairless dogs. Different breeds of hairless dogs such as the Mexican and Peruvian Hairless Dogs or the Chinese Crested Dog have been bred by dog fanciers for many centuries. These dogs have a sparse hair coat and dentition abnormalities similar to *EDA* mutant dogs. However, in contrast to *EDA* mutant dogs, the eccrine glands in the above mentioned hairless dogs breeds are normal. We identified a mutation in the gene encoding the transcription factor FOXI3 as causative for the ED phenotype in hairless dogs. The phenotypic similarities between *EDA* and *FOXI3* mutants suggest that FOXI3 is somehow involved in the ectodysplasin signaling pathway. However, the precise role of FOXI3 in ectodermal development has not yet been completely clarified.

